# Anti-tumor characteristics of radiation-induced tumor-infiltrating neutrophils

**DOI:** 10.1186/2051-1426-2-S3-P162

**Published:** 2014-11-06

**Authors:** Tsuguhide Takeshima, Ellen Vitetta, Raquibul Hannan

**Affiliations:** 1 Univ. of Texas Southwestern Medical Center, Dallas, TX, USA

## 

**Figure 1:**
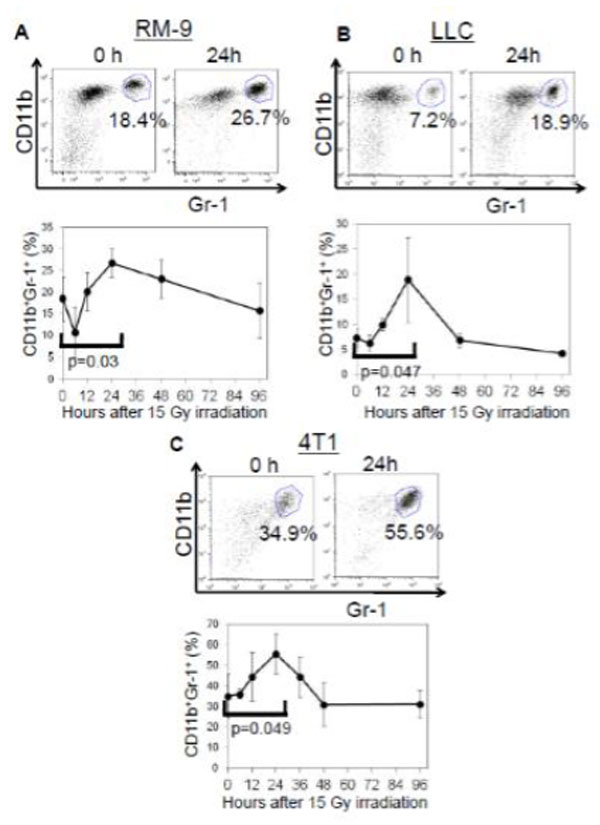
**CD11b^+^Gb-1^+^ cells increase in tumor tissue 24 hour after tumor irradiation**. RM-9- (a) LLC- (B) and 4T1- (C) bearing mice (n = 5) were sacrificed at different times after 15 Gy irradiation to the tumor on the left hind leg and the tumor tissues were analyzed for neutrophils (CD11b^+^Gr-1^+^ cells) by flow cytometry. The results show a significant increase in neutrophilic infilitration 24-48h after tumor irradiation.

**Figure 2:**
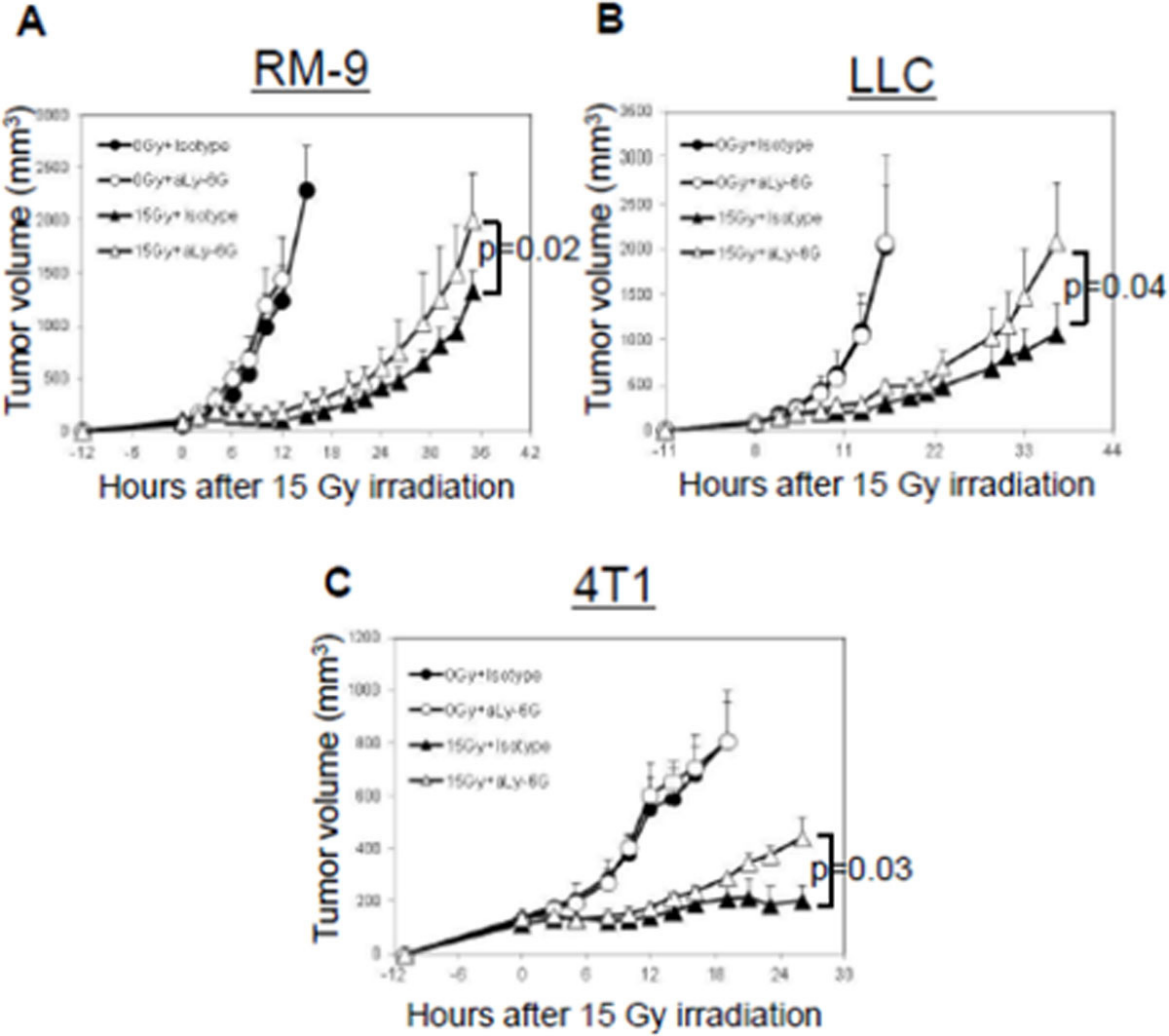
**Radiation-induced CD11b+Gr1+ cells impact therapeutic response of radiation**. RM-9- (A), LLC (B), and 4T1- (C) bearing mice were treated with i.p.injection of anti-Ly6G mAb (▲ and Δ) for neutrophil depletion or Isotype control (° and •) prior to irradiation (• and ▲) and compared to unirradiated (° and Δ) control tumor grafts. The results show a significant decrease in radiation therapeutic response with the depletion of neutrophils.

**Figure 3:**
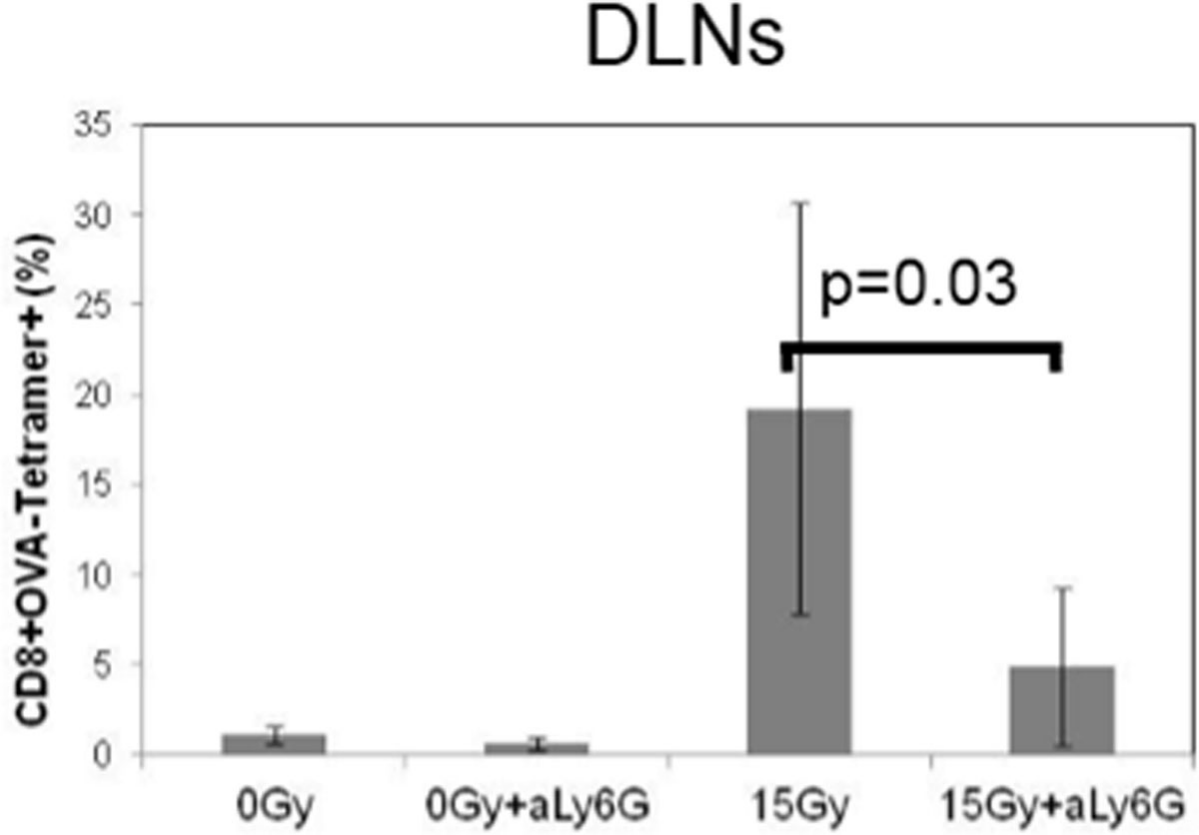
**Dependence of tumor-specific immune response on early tumor neutrophilic infiltration after irradiation**. RM-9-OV-bearing mice were irradiated focally with 15 Gy. DLN were harvested 9 days after irradiation and the percentage of tumor-specific CTL was quantified flow cytometry after staining with OVA tetramer and CD8. The results show a significant decrease in OVA tetramer+CD8+ cells with neutrophilic depletion.

It is now evident that interactions between tumor cells and host tissue stoma play a key role in tumor progression. Understanding the composition of the stromal cells in the tumor microenvironment immediately after tumor irradiation might be an important first step in understanding immunomodulation by radiation therapy. To explore this, we harvested tumor masses, draining lymph nodes (DLNs), spleens, and peripheral blood mononuclear cells (PBMCs) between 6h-96h after a single 15 Gy dose of focused irradiation of RM-9 mouse prostate tumor grafts growing in the hind leg of syngeneic C57BL/6 mice. Subpopulations of lymphocytes and granulocytes (CD4+, CD8+, CD4+CD25+, CD11c+, CD11b+Gr-1+mid, and CD11b+Gr-1+high cells) were analyzed by flow cytometry. We have previously reported that there is an infiltration of CD11b+Gr-1+high neutrophils into the tumor that reached a peak within 24-48 h after tumor irradiation (Fig. 1A). To investigate the generality of this phenomenon, the lung cancer cell line LLC and the breast cancer cell line 4T1 was implanted in their respective syngeneic hosts; C57BL/6 and BALB/c mice. In both models neutrophilic infiltration was observed at 24-48h after tumor irradiation (Fig. 1B, C). To investigate the effect of neutrophils on tumor growth, we compared the tumor sizes in mice treated with the neutrophil-depleting anti-Ly-6G mAb to those of mice treated with an isotype-matched control antibody. The therapeutic effect of irradiation was significantly attenuated in all three tumor models when neutrophils were depleted (Fig. 2). To evaluate the impact of the neutrophilic infiltration on tumor-specific immune responses, we generated OVA gene-transfected RM-9 and RM-9-OVA-tumor-bearing mice for detecting OVA-specific CTL. Cohorts of RM-9-OVA-tumor-bearing mice with and without neutrophil depletion by anti-Ly-6G mAb were irradiated focally with 15 Gy. Nine days after irradiation, DLNs were harvested and started to culture under IL-2 and IL-12 with irradiated RM-9-OVA cells. Five days later, OVA-specific CTLs were quantified by flow cytometry after staining with OVA-tetramer. The result (Fig. 3) shows an increased frequency of CD8+OVA-tetramer+ cells in irradiated tumor-bearing mice and a decrease in CD8+OVA-tetramer+ cells in the neutrophil-depleted mice. Furthermore, this immune response correlates with the delay in tumor growth. Therefore, neutrophil infiltration may be an early but important step in the radio-immunomodulation of tumor that is important for the initiation adaptive tumor-specific immune responses. Current endeavors are focused on delineating the steps involved in the generation of tumor-specific immune response from radiation-induced neutrophilic infiltration.

